# Unusual presentation and delayed diagnosis of cardiac angiosarcoma

**DOI:** 10.1186/s13019-024-02555-8

**Published:** 2024-03-28

**Authors:** Salman Zaheer, Alice L. Zhou, John M. Gross, Ahmet Kilic

**Affiliations:** 1 Division of Cardiac Surgery, Department of Surgery, The Johns Hopkins Hospital, Baltimore, MD USA; 2 Bone and Soft Tissue Pathology, The Johns Hopkins Hospital, 1800 Orleans Street, Zayed Tower Suite 7107, Baltimore, MD 21287 USA

**Keywords:** Cardiac tumor, Pseudoaneurysm, Angiosarcoma, Tumor resection, Case report

## Abstract

**Background:**

Primary cardiac angiosarcomas are very rare and present aggressively with high rates of metastasis. Given the poor prognosis, particularly once disease has spread, early diagnosis and multidisciplinary treatment is essential.

**Case presentation:**

We present the case of a 46-year-old male who presented with chest pain, intermittent fevers, and dyspnea. Workup with computed tomography scan and transesophageal echocardiography demonstrated a right atrial pseudoaneurysm. Given the concern for rupture, the patient was taken to the operating room, where resection of the pseudoaneurysm and repair using a bovine pericardial patch was performed. Histopathology report initially demonstrated perivascular lymphocyte infiltrate. Six weeks later, the patient represented with chest pain and new word finding difficulty. Workup revealed multiple solid lung, pericardial, brain, and bone nodules. Eventual biopsy of a cardiophrenic nodule demonstrated angiosarcoma, and rereview of the original pathology slides confirmed the diagnosis of primary cardiac angiosarcoma.

**Conclusions:**

Primary cardiac angiosarcomas are often misdiagnosed given the rarity of these tumors, but early diagnosis and initiation of treatment is essential. The unique presentation of our case demonstrates that clinical suspicion for cardiac angiosarcoma should be maintained for spontaneous pseudoaneurysm originating from the right atrium.

## Background

Primary cardiac angiosarcomas are very aggressive tumors that often present after metastatic spread. Given the aggressive nature of the disease, early diagnosis is essential. We describe the case of a patient with primary cardiac angiosarcoma who initially presented with a right atrial pseudoaneurysm.

## Case presentation

We present a case of 46-year-old male with no pertinent past medical history who presented to the emergency department with ongoing chest pain for 9 days associated with intermittent fevers and shortness of breath. On examination, the patient had a heart rate of 88 beats per minute and blood pressure of 143/72 mmHg. There were no audible murmurs or rubs on auscultation. He endorsed ongoing chest pain and shortness of breath.

**Fig. 1:**
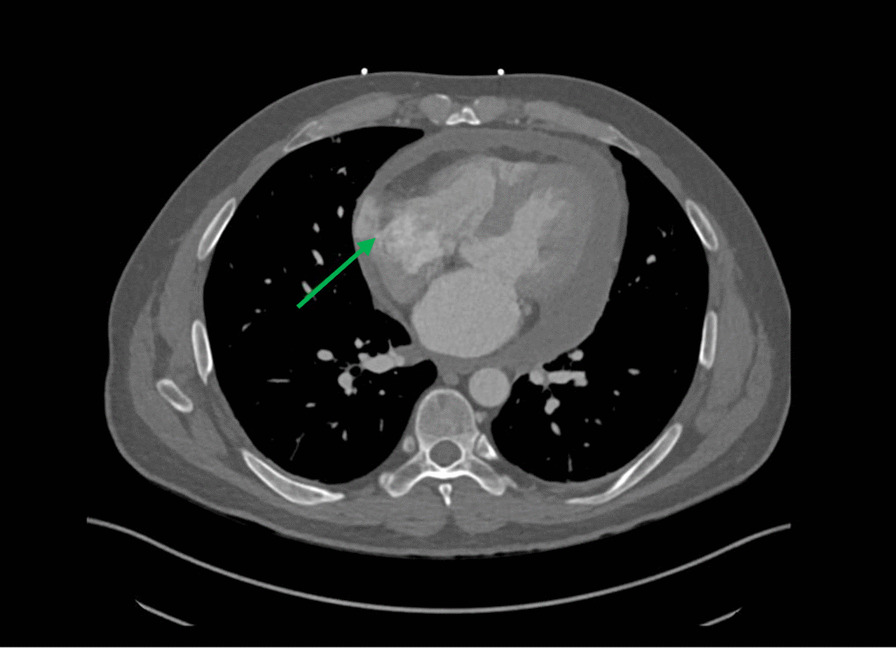
Computed tomography angiogram of the chest. Computed tomography angiogram showing a right atrial pseudoaneurysm. Neck of the pseudoaneurysm is shown by the green arrow. Attenuation of the pericardial fluid was 28.4 Hounsfield units

**Fig. 2:**
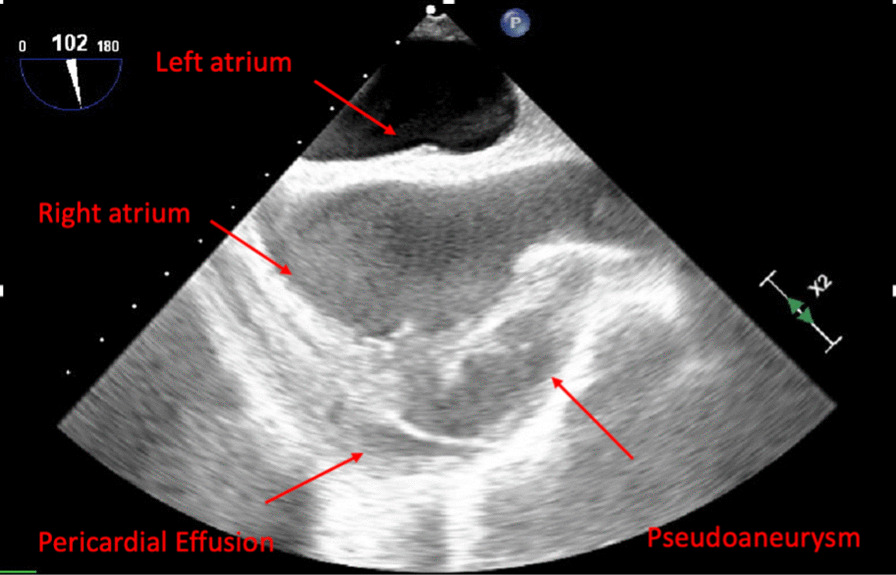
Transesophageal echocardiogram of pseudoaneurysm. Pseudoaneurysm with pericardial effusion redemonstrated on transesophageal echocardiogram arising from right atrium

The patient underwent contrast computed tomography (CT) angiography pulmonary embolism with pulmonary arterial phase timing that showed no evidence of pulmonary embolism, but demonstrated a moderate sized pericardial effusion with high density concerning for hemopericardium, along with an outpouching on the right atrial wall with a narrow neck concerning for a pseudoaneurysm (Fig. 1). Further workup, including lab work and an electrocardiogram, were unremarkable. Transesophageal echocardiogram (TEE) redemonstrated the pericardial effusion and right atrial pseudoaneurysm (Fig. 2).

**Fig. 3:**
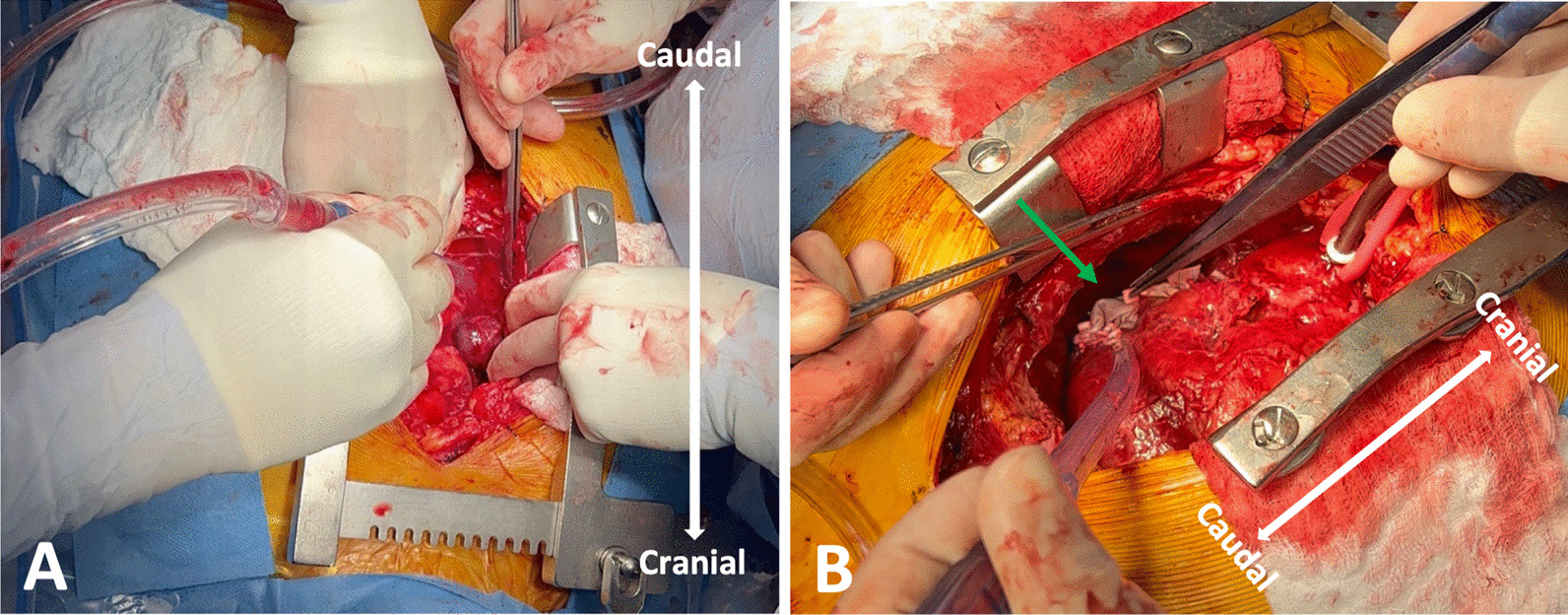
Intra-operative photos of pseudoaneurysm. Pseudoaneurysm demonstrated by green arrow. **A** Blue arrow indicates pseudoaneurysm. **B** Green arrow indicates bovine pericardial patch used to repair defect in atrial wall after pseudoaneurysm resection

The case was discussed at the multidisciplinary cardiac conference and the decision was made to take the patient to the operating room given his ongoing symptoms as well as concern for hemopericardium and rupture of pseudoaneurysm into the pericardial space. Cardiac magnetic resonance imaging (MRI) and positron emission tomography (PET) CT were not performed given time-sensitive nature of the case and the desire for tissue diagnosis. The operation was performed via median sternotomy with cardiopulmonary bypass. A pericardial effusion and dense pericardial adhesions were found intraoperatively. After meticulous lysis of adhesions, central cannulation was performed, and the aorta and right atrium were cannulated. Right atrial pseudoaneurysm was identified (Fig. 3) and excised with the surrounding atrial wall. The defect in the atrial wall was closed using a bovine pericardial patch. Full exploration of the chest revealed no other lesions. The resected specimen was sent for pathology to try to establish tissue diagnosis and the patient was closed. The patient’s post-operative course was unremarkable, and he was discharged home on post operative day 4. Operative cultures showed no growth. Pericardial fluid cytopathology demonstrated abundant mixed inflammation with eosinophils, reactive mesothelial cells, histiocytes, and lymphocytes, with no malignant neoplasm identified. Tissue histopathology demonstrated granulation tissue, perivascular lymphocyte infiltrate, dense fibrous tissue, focal calcification, and organizing fibrin thrombus, but no evidence of malignancy.

**Fig. 4:**
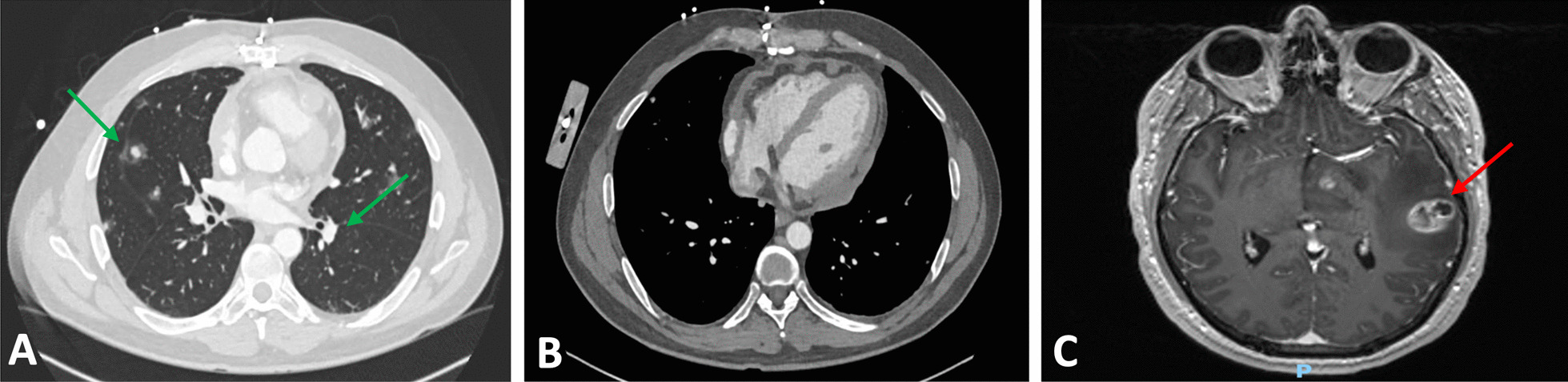
Imaging findings on representation. **A** Computed tomography (CT) of chest showing multiple lung nodules (green arrows). **B** CT of chest showing nodular appearance of pericardial effusion and outpouching of the right atrium. **C** Head magnetic resonance imaging (MRI) demonstrating peripherally enhancing lesions (red arrow)

Six weeks postoperatively, the patient presented to emergency department with chest pain, new word finding difficulty, clumsiness, gait instability, and intermittent low-grade fevers. A CT chest scan showed multiple halo-like solid lung, pericardial, and bone nodules (Fig. 4A, B). The CT head scan showed 1–1.5 cm left anterior thalamic and left temporal lobe hemorrhagic lesions. Magnetic resonance imaging of the brain demonstrated multiple enhancing lesions in the left temporal lobe, left anterior thalamus, and right parietal lobe (Fig. 4C). Together, the findings were suspicious for hemorrhagic metastasis, invasive fungal infection (such as invasive aspergillosis), or septic emboli. Given the concern for metastatic process, a biopsy of a lung nodule was performed and showed focal organizing exudative and fibrotic pneumonia negative for carcinoma. A lumbar puncture was also performed and showed no malignant neoplasm. Given the repeated surgical specimens that were negative for malignancy, an extensive infectious and rheumatologic workup was pursued. He was discharged with outpatient infectious disease follow-up, as the most likely diagnosis on the differential was thought to be invasive fungal infection and cultures would take several weeks to result.

**Fig. 5:**
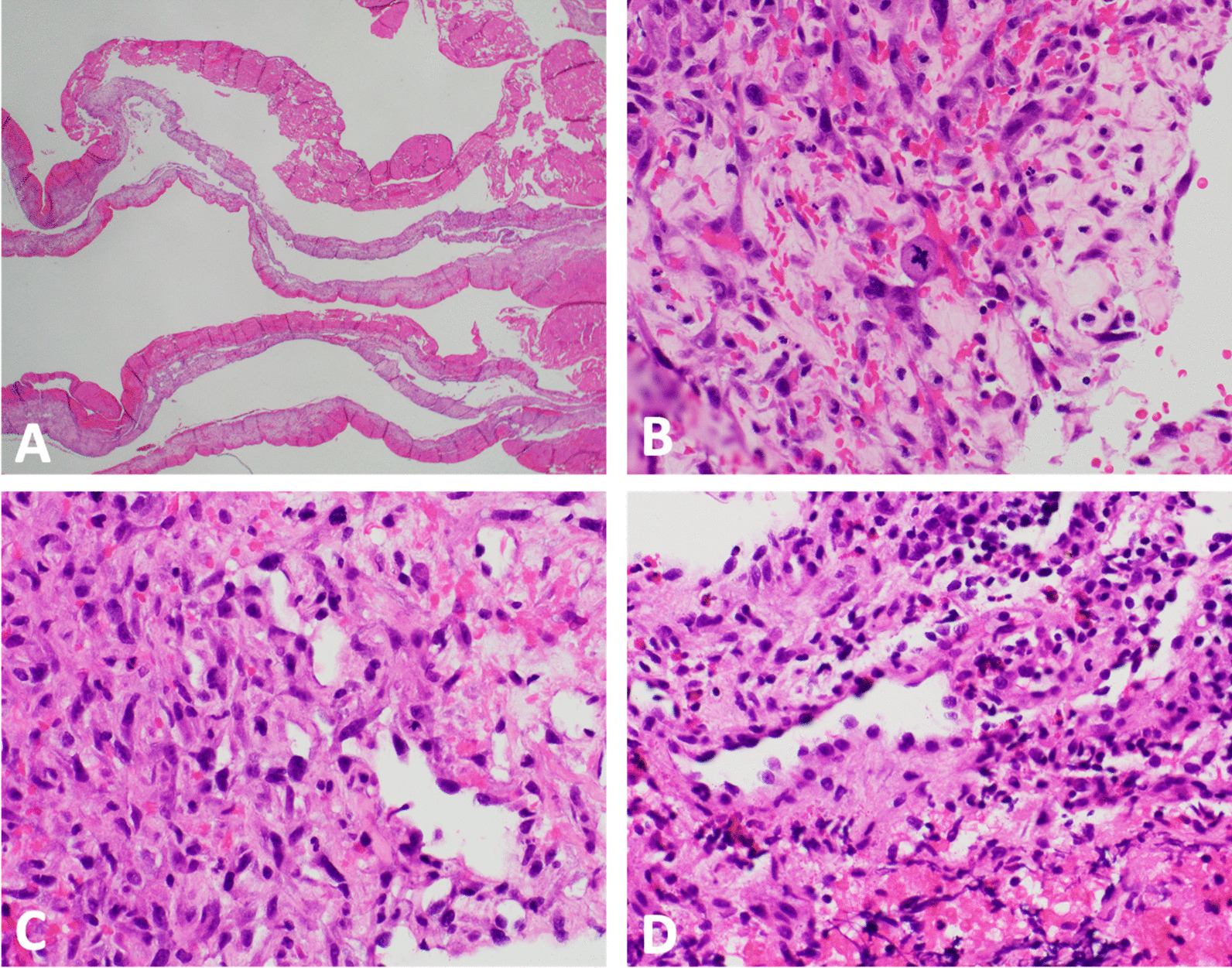
Pathology findings demonstrating angiosarcoma. **A** low power magnification (× 12.5) hematoxylin and eosin staining of pseudoaneurysm reveals fibrin and organizing hematoma with a variably cellular fibrous cyst wall. **B** High power magnification (× 400) of pseudoaneurysm reveals scattered enlarged pleomorphic tumor cells with rare atypical mitoses in the background of abundant reactive granulation tissue. **C** Cardiophrenic nodule (× 400) shows similar atypical cells with unequivocal anaplasia and poorly-formed vascular lumina. **D** Cardiophrenic nodule (× 400) shows malignant endothelial cells with endothelial hobnailing into the vascular lumen

He represented one week later with returned right-sided chest pain, intermittent headaches, and abdominal pain. Repeat CT scan demonstrated increased number of pulmonary and pericardial nodules. Given the patient’s diagnostic dilemma and ongoing symptoms, a cardiophrenic nodule was biopsied with image guidance. Pathology demonstrated atypical cells, anaplasia, and poorly-formed vascular lumina, suggestive of angiosarcoma (Fig. 5).

The patient’s tissue biopsy from his initial right atrial pseudoaneurysm resection was retrospectively reviewed and similar atypical cells were noted in the background of granulation tissue and organizing thrombus (Fig. 5). A diagnosis of primary cardiac angiosarcoma was made. The patient is currently undergoing chemotherapy with weekly paclitaxel for metastatic cardiac angiosarcoma.

## Discussion and conclusions

Primary tumors of the heart are very rare and are identified in only 1 in 500 cardiac surgical cases. Of these, malignant tumors make up only 25%, the majority (95%) of which are cardiac sarcomas [1]. Angiosarcomas are the most common histologic subtype of cardiac sarcomas (30%) and are very aggressive in nature with high rates of metastasis [2].

Cardiac angiosarcomas more frequently present in males (2–3:1 male:female ratio) and most commonly arise from the right atrium [3]. Patients often present with nonspecific symptoms such as chest pain, dyspnea, weight loss, and fatigue [1]. Later in the disease course, patients may present with more specific symptoms, such as pericardial effusion, due to local infiltration into the myocardium and neighboring structures. Over half of patients present with metastatic disease, with spread to the lungs or pleura being most common [3]. Imaging can aid in the diagnosis of cardiac angiosarcomas. CT scan often shows a large multilobular mass that comprises the majority of the right atrium [4]. Transthoracic echocardiography (TTE) often shows a large echogenic mass with poorly defined borders and can be used to understand the size and location of the mass, understand its relation to other structures, and determine if cardiac function is affected [5]. Magnetic resonance imaging (MRI) can be used to differentiate between thrombus and tumor [6]. Histopathologically, angiosarcomas are difficult to diagnose due to the cellular heterogeneity. Common tumor markers include CD34, CD31, and factor VIII [1].

Due to the aggressive nature of this cancer, early diagnosis is essential. Our case represented a diagnostic challenge and initially presented on imaging as a right atrial pseudoaneurysm with a narrow neck, as opposed to a large multilobular mass with broad-based attachment typically described for cardiac angiosarcoma [4, 5]. Diagnosis was further delayed by the challenging histologic presentation in the context of low clinical suspicion, with malignant cells being obscured by extensive fibrin, granulation tissue, and organizing thrombus. Given these difficulties, diagnosis was not made until after distant metastatic spread and a cardiophrenic metastatic lesion was biopsied.

Given the rarity of this tumor and high mortality rate, there is no standardized approach to treatment. Without surgical resection, average survival is about 4 months [1]. The mainstay of treatment involves complete surgical resection, with a median post-operative survival of about 14 months [1, 3]. However, achieving complete resection with negative margins is often difficult given the diagnostic delay and proximity to vascular structures and propensity for distant microscopic spread at time of diagnosis. Chemotherapy and radiation are often used in the treatment either as neoadjuvant or adjuvant therapy, but the exact role of these modalities remains unknown [7, 8]. Overall, a multidisciplinary approach involving local resection, systemic chemotherapy, and radiation therapy seems to be the most favorable strategy.

Primary cardiac angiosarcomas are often misdiagnosed given the rarity of these tumors. Cardiac angiosarcoma presenting initially on imaging as either a right atrial pseudoaneurysm or a right coronary artery aneurysm has only been reported in a handful of case reports [9–14]. The unique presentation of this case demonstrates that clinical suspicion for cardiac angiosarcoma should be maintained for spontaneous pseudoaneurysm originating from the right atrium. With an aggressive disease course, early diagnosis is vital and allows for multidisciplinary care with medical and radiation oncology to offer patients the best chances of survival.

## Data Availability

Not applicable.

## Abbreviations

CT: Computed tomography
MRI: Magnetic resonance imaging
PET: Positron emission tomography
TEE: Transesophageal echocardiogram
TTE: Transthoracic echocardiography
